# AgriPest-YOLO: A rapid light-trap agricultural pest detection method based on deep learning

**DOI:** 10.3389/fpls.2022.1079384

**Published:** 2022-12-16

**Authors:** Wei Zhang, He Huang, Youqiang Sun, Xiaowei Wu

**Affiliations:** ^1^Institute of Physical Science and Information Technology, Anhui University, Hefei, China; ^2^Institute of Intelligent Machines, Hefei Institute of Physical Science, Chinese Academy of Sciences, Hefei, China; ^3^Technology Research and Development Center, Anhui Zhongke Intelligent Sence Industrial Technology Research Institute, Wuhu, China

**Keywords:** agricultural pest detection, light trap, YOLO, attention mechanism, multi-scale

## Abstract

Light traps have been widely used for automatic monitoring of pests in the field as an alternative to time-consuming and labor-intensive manual investigations. However, the scale variation, complex background and dense distribution of pests in light-trap images bring challenges to the rapid and accurate detection when utilizing vision technology. To overcome these challenges, in this paper, we put forward a lightweight pest detection model, AgriPest-YOLO, for achieving a well-balanced between efficiency, accuracy and model size for pest detection. Firstly, we propose a coordination and local attention (CLA) mechanism for obtaining richer and smoother pest features as well as reducing the interference of noise, especially for pests with complex backgrounds. Secondly, a novel grouping spatial pyramid pooling fast (GSPPF) is designed, which enriches the multi-scale representation of pest features *via* fusing multiple receptive fields of different scale features. Finally, soft-NMS is introduced in the prediction layer to optimize the final prediction results of overlapping pests. We evaluated the performance of our method on a large scale multi pest image dataset containing 24 classes and 25k images. Experimental results show that AgriPest-YOLO achieves end-to-end real-time pest detection with high accuracy, obtaining 71.3% mAP on the test dataset, outperforming the classical detection models (Faster RCNN, Cascade RCNN, Dynamic RCNN,YOLOX and YOLOv4) and lightweight detection models (Mobilenetv3-YOLOv4, YOLOv5 and YOLOv4-tiny), meanwhile our method demonstrates better balanced performance in terms of model size, detection speed and accuracy. The method has good accuracy and efficiency in detecting multi-class pests from light-trap images which is a key component of pest forecasting and intelligent pest monitoring technology.

## 1 Introduction

Agriculture development has been limited by many factors, especially the frequent occurrence of crop pests, which has seriously affected agricultural production. Crop pest control has always been a priority issue for agricultural producers, and plays an important role in guaranteeing world food security and normal economic development. Chemical pesticides have been an important weapon in the fight against pests for a long time, and agricultural workers need to configure pesticide species and doses according to the population dynamics of pests in real-world scenarios (Wang Q.-J. et al., 2020), to prevent food safety and environmental pollution caused by pesticide overuse. In traditional agricultural production, the way to obtain pest population dynamics in the field mainly relies on manual surveys. However, this approach is labor-intensive and has some obvious drawbacks: inefficiency, subjectivity, error-prone, and lagging information. For the sake of food security and yield, it is desirable to develop an automatic pest monitoring method with high efficiency and accuracy.

Fortunately, with the development of information science, new problem-solving ideas are offered (Li X. et al., 2021), namely precision agriculture in which information technology is integrated with agricultural production. In this modern production context, capturing images of pests using light traps and then counting and evaluating them by automated pest detection methods based on computer vision technology has become a mainstream research hotspot (Jiao et al., 2022b). From the algorithmic viewpoint, early research has focused on machine learning frameworks, can be summarized in two main steps: pest-related information extraction from images as feature vectors and machine learning classifiers for classification. Xie et al. (2015) adopted a sparse-coding histogram with multiple feature modalities to represent pest images, and multiple-kernel learning (MKL) techniques were used to fuse multiple features, to form a multi-class classifier for the identification of 24 classes of pests. Yao et al. (2012) exploited the radial basis kernel function to extract features such as color, shape, and texture of each pest in the pest image which was taken as input to the Support Vector Machine (SVM) for the identification of four species of Lepidoptera rice pests. Wen et al. (2009) proposed a method based on invariant local features for the identification of common pests in orchards, and compared the identification results of multiple classifiers on five pest datasets. On the whole, the above machine learning based pest identification methods have achieved good performance to some extent, but most of the work of traditional algorithms aims at solving the part of the pest detection issue, the classification problem, while few focus on the more challenging localization problem. Besides, their performance is overly dependent on the applicability of manually extracted features to the target, making it hard to be applied in practical scenarios.

With the upgrading of hardware and software, the rapid development of deep learning has been driven. Compared with traditional machine learning, deep learning techniques based on Convolutional Neural Network (CNN) have the potential to become an effective approach to solve challenging tasks in intelligent pest monitoring because of their efficient feature self-learning and self-organizing strategy power (Xie et al., 2021). Object detection techniques based on deep learning employ one-stage and two-stage strategies. To improve the pest identification accuracy, two-stage algorithms with higher accuracy but slower detection speed than one stage algorithms are usually used to monitor agricultural pests (Jiao et al., 2022a; Zhao et al., 2022). For example, for detecting densely distributed aphids in the field, Li et al. (2022) proposed a multi-branch convolutional neural network (Mb-CNN) based on density map, which extracts different scale feature maps by multiple branches of the model for generating aphid density maps, and finally estimating aphid numbers. However, this method has specificity and unable to be transferred to the detection of multiple categories of agricultural pests. Zhao et al. (2022) designed an improved Faster RCNN (Ren et al., 2017) model based on multi-scale feature fusion for detecting diseases of strawberries in natural environments and achieved 92.8% mAP, but the larger computational demands led to reduced detection speed. To gather population information of tiny pests in agricultural greenhouses, Li W. et al. (2021) developed an end-to-end model based on Faster RCNN, TPest-RCNN for detecting whitefly and thrips in sticky trap images. Liu et al. (2022) proposed a two-stage CNN model based on global activated feature pyramid network (GaFPN) for detecting six tiny pests in field scenes. Through global activated module (GAM), the channel and location attention are extracted at different layers of the feature pyramid in a parallel manner to generate the selected weight, to balance the feature pyramid network and solve some obstacles of tiny pest detection. However, when the pest has a complex background, GaFPN fails to filter out the pest features, resulting in sub-optimal detection results. Zhang et al. (2022) improved the YOLOv4 network (Bochkovskiy et al., 2020) *via* attention mechanism and contextual information to detect pest regions in maize with different growth cycles. In addition to targeting specific agricultural pests, some studies have reported advances in detection methods for multi-class agricultural pests. Wang Q.-J. et al. (2020) established a standardized dataset consisting of 24 classes of typical agricultural pest images, and reported the detection results of four advanced deep convolutional neural networks, among which YOLOv3 (Redmon and Farhadi, 2018) had the best performance, yet these methods have not taken into account the specificity of agricultural pest images. Jiao et al. (2020) proposed an anchor-free region convolutional neural network (AF-RCNN) that could detect 24 types agricultural pests by an end-to-end way. AF-RCNN has poor performance in detecting some pests with few training samples. Tang et al. (2021) proposed a real-time detection model Pest-YOLO based on YOLOv4 and improved CNN for agricultural pest image data mining, and several comparative experiments had shown the performance of Pest-YOLO. Wang et al. (2022) designed an efficient channel and spatial attention network (ECSA-Net), and an optimized image pre-processing algorithm, Sparse Mask Super-resolution (SMSR), to construct an automatic pest identification framework, that was applied to detect ten pests in the natural environment. But it is not applicable to real-time detection. Jiao et al. (2022b) developed a two-stage model, adaptive feature pyramid network (AFFP-Net), and experimentally showed that 77.0% accuracy was obtained on a large pest dataset containing 21 pest classes. While the network is computationally intensive and difficult to apply to mobile terminals. Although promising, there are various scale variations, dense distributions and complex background samples in the light-trap pest dataset, and pest detection methods in agriculture still have the necessity to be optimized. Besides, these high-performance CNN models have more parameters and large computation, which are not conducive to the real-time monitoring of agricultural pests and limit the application to mobile devices. Hence, the above methods still fail to satisfy the practical needs of real-time pest monitoring.

YOLOv5 (Glenn, 2020) is a state-of-the-art one-stage deep learning framework that achieves optimal performance for real-time object detection. However, as for agricultural pest detection, it lacks the ability to extract key features from pest images containing large background noise and dense distributions, and struggles to capture detailed features for pest instances belonging to few samples and extreme small size. This motivates the development of an improved YOLOv5 model for monitoring agricultural pests that is to achieve a balance between detection speed, accuracy and model size. Firstly, to strengthen the discriminative and representative ability of the network for pest features in complex backgrounds, coordinate and location attention (CLA) module is designed to be fused into the backbone. The channel attention map is decomposed into two parallel one-dimensional feature vectors, so that location information is embedded in the channel attention, and combined with local channel attention to filter some noise, some useful pest features dominate with more discriminative cues. Then, in view of the scale variation of pests, we propose a grouping spatial pyramid pooling fast (GSPPF) module to further augment the multi-scale representation of pest features through fusion multiple receptive fields of different scale features. Finally, a post-processing algorithm soft-NMS is introduced in the prediction stage to improve the detection accuracy of the network for overlapping pests. The improved model was comprehensively evaluated through extensive comparative experiments. Experimental results show that the proposed lightweight detection models, AgriPest-YOLO, outperforms other advanced detection methods, which improves detection accuracy while maintaining detection efficiency.

The remainder of this paper is organized as follows. Section 2 contains a brief introduction of the material used in this study. Section 3 elaborates on the proposed pest detection method. Section 4 reports the comparison experiment results and analysis. Finally, we conclude our work and discuss future work in section 5.

## 2 Materials

**Figure 1:**
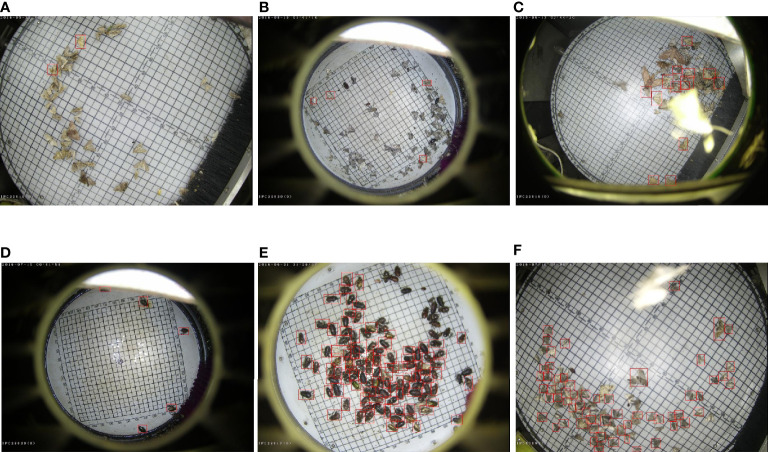
Some examples of pest images in Pest24, the red boxes are the pests that need to be predicted; pest detection from light traps is affected by various factors, **(A-C, E, F)**: non-target pest background, **(C, F)**: accidental occlusion, **(B, D)**: oversized non-target background, **(D)**: inflection points caused by light, **(E, F)**: dense distribution.

We evaluate our methods on a pest dataset called Pest24 (Wang Q.-J. et al., 2020). Pest24 is a large-scale, multi-target agricultural pest standardized dataset where all images were collected by automated pest trapping devices in real field environments. Pest24 has the following significant features: (1) a large amount of data. The basic information in the dataset is shown in **Table 1**, that involves 24 categories of typical pests of field crops and contains 25378 images, including 12701 training images, 5077 validation images and 7600 test images, all of which have a resolution of 800×600 pixels. (2) Complex background. The complexity of the wild environment brings a lot of uncontrollable factors resulting in the appearance of irrelevant noise in the pest images. As shown in **Figure 1**, (a): The non-target pest has similar appearance with the target pest. (b): The non-target background region is too large and the relative size of the target pest is reduced. (c): Shadows, occlusion. (d): Inflection points caused by light. (3) Pest scale, the pest size is very small and the relative scale is mainly distributed in (0, 0.01), other than that, the pest scale is extremely variable, up to around 1600. (4) Unbalanced distribution of sample categories. The images and the number of instances of each category of pests are shown in **Table 1**, which can be seen that Pest24 belongs to the long-tailed distribution dataset. (5) Dense distribution and target adhesions. (6) Interclass similarity and intraclass variation. Note that more than one kind of complex background may appear in a single image, such as the appearance of non-target pest background and occlusion in **Figures 1C, D**, which is an image with oversized non-target background in addition to the presence of reflected light points. As can be observed from **Figures 1E, F**: dense distribution, target adherence and complex background are not present alone in a single image. In conclusion, these characteristics of the dataset pose a great challenge for the accurate detection of pests.

**Table 1:** Description of the 24 categories of pest information from Pest24 dataset, including the number of images, instances and Scale for each category.

Index	Pest name	Scale	Number of images	Number of instances	Index	Pest name	Scale	Number of images	Number of instances
1	*Rice planthopper*	0.034	316	1511	15	*Spodoptera cabbage*	0.42	1707	2302
2	*Rice Leaf Roller*	0.123	944	1240	16	*Scotogramma trifolii Rottemberg*	0.28	3223	4679
3	*Striped rice borer*	0.186	454	1285	24	*Yellow tiger*	0.398	1388	1686
5	*Armyworm*	0.394	3828	8880	25	*Land tiger*	0.639	369	475
6	*Bollworm*	0.281	9049	28014	28	*Eight-character tiger*	0.441	154	168
7	*Meadow borer*	0.226	5526	16516	29	*Holotrichia oblita*	0.334	90	108
8	*Athetis lepigone*	0.13	7520	30339	31	*Holotrichia parallela*	0.255	3111	11675
10	*Spodoptera litura*	0.458	1588	1951	32	*Anomala corpulenta*	0.249	5228	53347
11	*Spodoptera exigua*	0.138	3614	7263	34	*Gryllotalpa orientalis*	0.95	3629	6528
12	*Stem borer*	0.277	1357	1804	35	*Nematode trench*	0.32	118	167
13	*Little Gecko*	0.57	2503	4279	36	*Agriotes fuscicollis Miwa*	0.114	1814	6484
14	*Plutella xylostella*	0.043	531	953	37	*Melahotus*	0.158	239	768

Scale represents the average relative scale (the ratio of the size of annotated bounding box to the size of original image).

**Figure 2:**
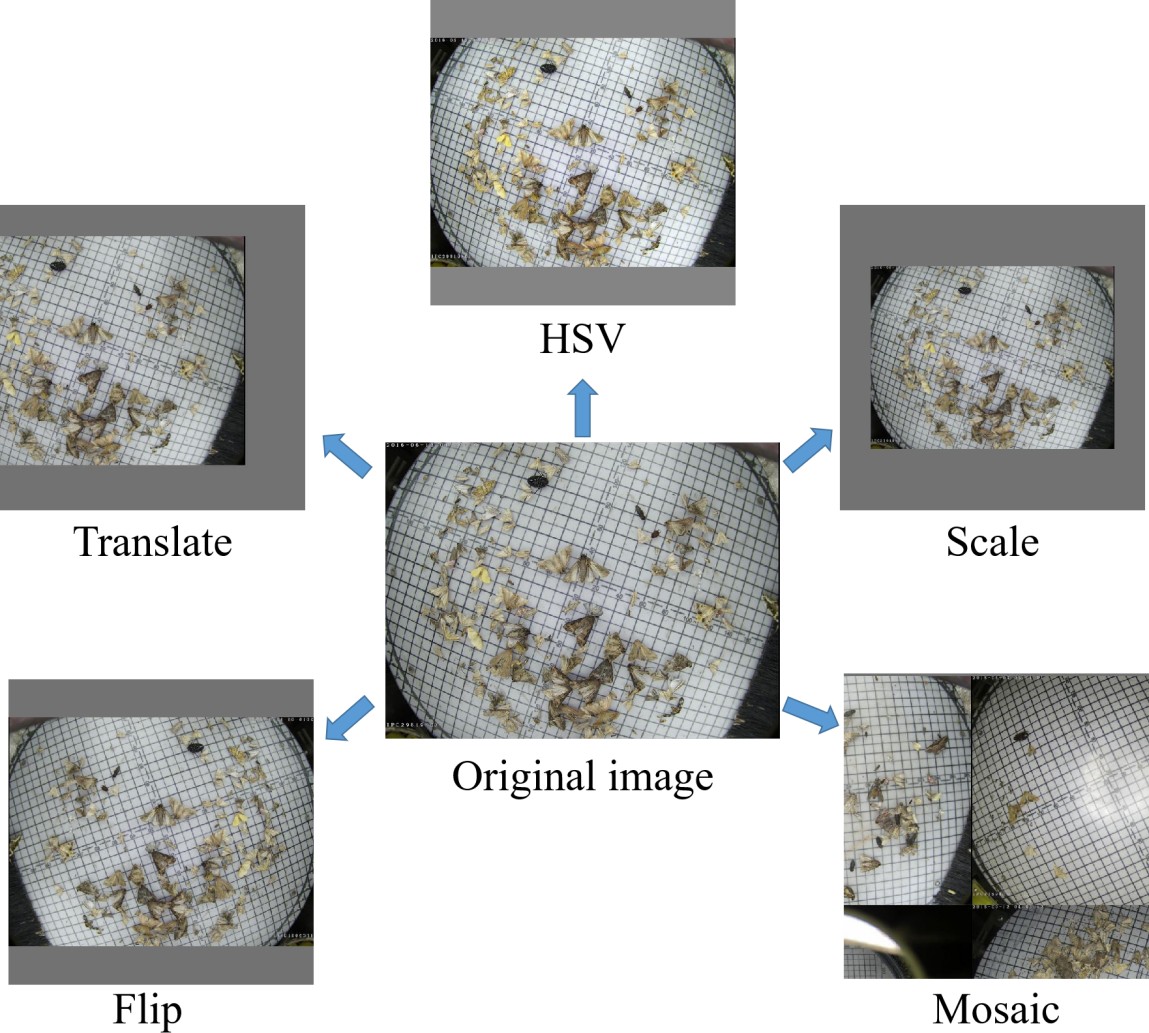
Examples of different data augmentation methods; HSV: color-space augmentation; Flip: the image is flipped with some probability; Translate: the image is translated with some proportion; Scale: the image is scaled within some range; Mosaic: the four training images are stitched together after random cropping.

Data has a substantial impact on deep learning. In order to enrich the diversity of training samples in the Pest24 dataset, improve model robustness and avoid overfitting. As shown in **Figure 2**, there were several online data augmentation methods adopted: (1) HSV: color-space augmentation (2) Filp: flipping with 50% probability (3) Translate: translation factor of 0.1 (4) Scale: the scaling factor is randomly picked between 0.5 and 1.5 (when the scaling factor equals to 1, the image size remains constant). (5) Mosaic data augmentation, blending four training images to improve the generalization ability of the model, which has helped to detect tiny pests. In the HSV color space augmentation method, the original image is converted from RGB color space to the color space consisting of three components: hue, saturation, and value, then the color transformation is performed by perturbing these three components in the HSV color space (perturbation coefficients are 0.03, 0.7, and 0.5, respectively), to enrich the color information of the training samples.

## 3 Methodologies

### 3.1 The proposed AgriPest-YOLO for pest detection

**Figure 3:**
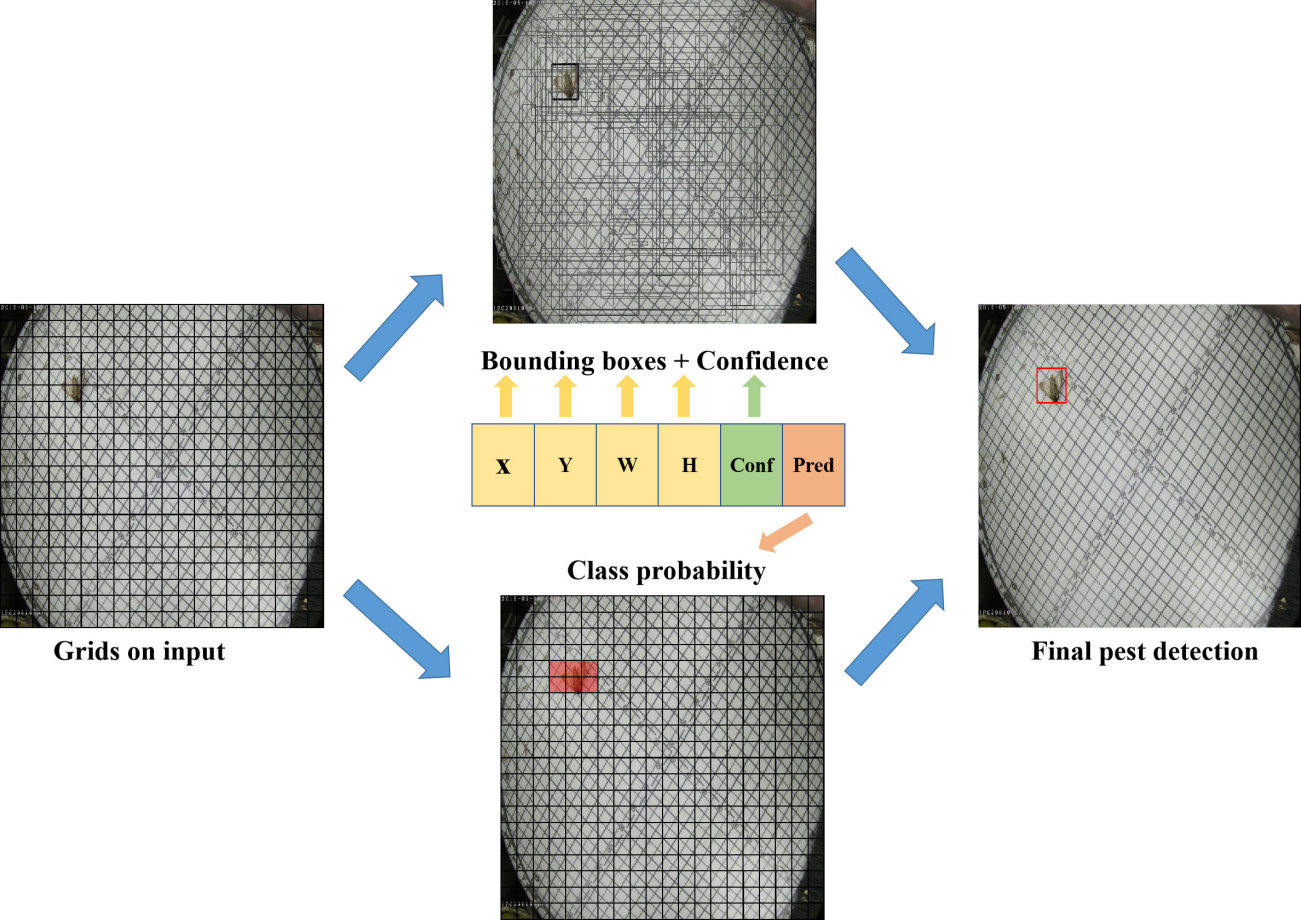
The principle of YOLO detection; X,Y are the center coordinates of the prediction bounding box; H, W are the length and width of the prediction bounding box; Conf, Pred are the confidence score and class probability, respectively.


YOLOv5 network is continuously optimized from YOLO series algorithm (Redmon and Farhadi, 2017; Redmon and Farhadi, 2018; Bochkovskiy et al., 2020; Chen et al., 2021), which is a typical one-stage object detection algorithm. Compared with two-stage target detection algorithms, such as the most widely used Faster RCNN, YOLO algorithm takes the whole image as input without generating proposal region, that greatly improves the detection speed and reduces the computational cost. The YOLO algorithm transforms the detection problem into a regression problem. The detection principle is shown in **Figure 3**, where the input image is divided into *S*×*S* grids, and each grid is responsible for detecting targets in which the central point falls within this grid, and then generating prediction bounding box information and confidence scores through regression. The prediction parameters consist of the prediction bounding box information (center coordinates: X, Y; length and width: W, H), the confidence score (Conf) and the probability of different classes (Pred). The confidence score is calculated as follows:

(1) $$Conf_{j}^{i}=P_{r}\left(Object\right)\times IoU_{pred}^{truth}$$

where $Conf_{j}^{i}$ denotes the confidence score of the i-th predicted bounding box of the j-th grid, and *P*_*r*_(*Object*) is 1 when the predicted bounding box contains pest and 0 vice versa. $IoU_{pred}^{truth}$ is the possible intersection over union (IoU) between the predicted bounding box and the target ground truth. After generating the prediction bounding boxes, the final prediction results are generated by the post-processing algorithm non-maximum suppression (NMS) filtering.

**Figure 4:**
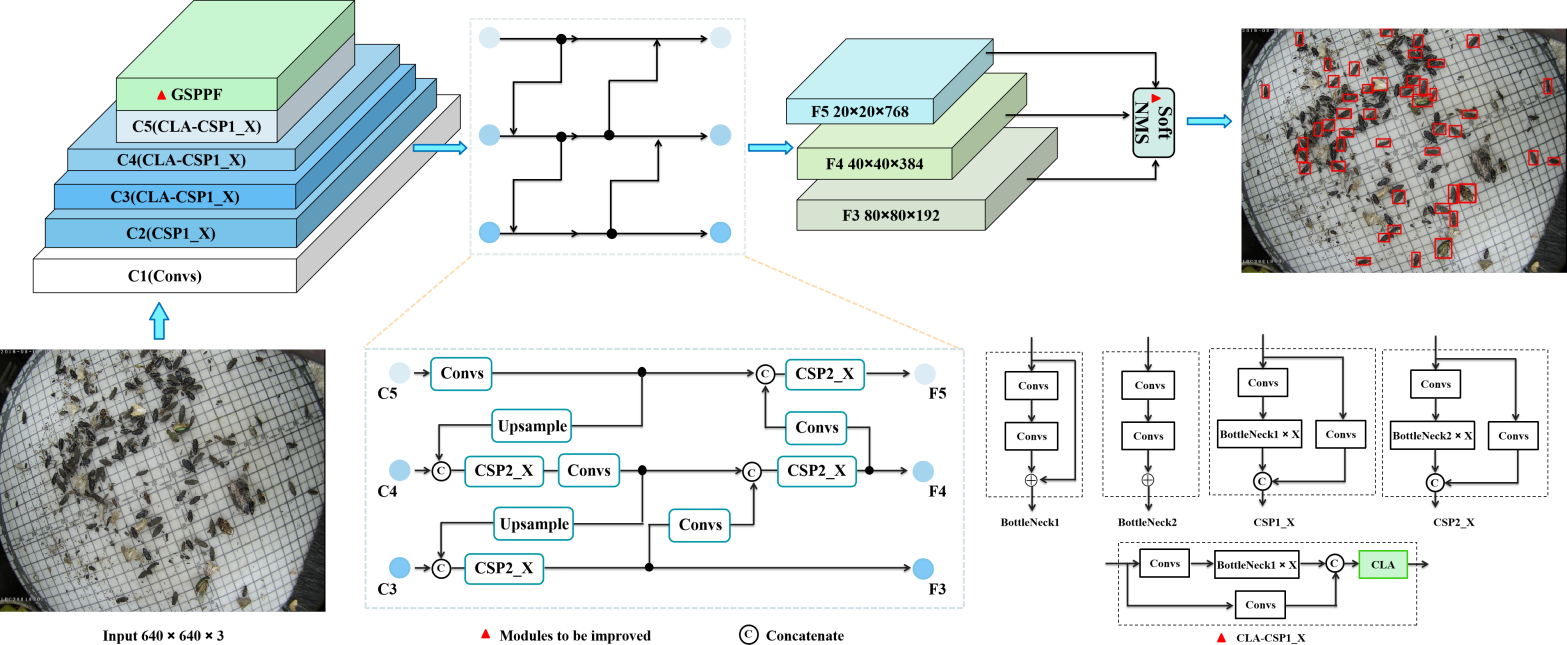
Pest detection network AgriPest-YOLO structure, consisting mainly of backbone network, GSPPF, neck and head.

The main components of YOLOv5 network include backbone network, SPPF, neck, and head. There are five versions of YOLOv5 depending on the depth and width of the network, including YOLOv5n, YOLOv5s, YOLOv5m, YOLOv5l, and YOLOv5x. The lightweight model YOLOv5s has the better overall performance among the five versions and meets the demand of real-time detection. However, it ignores the particularity of pest images, so we propose an improved YOLOv5s network, AgriPest-YOLO, for real-time pest detection. The overall structure is shown in **Figure 4**, and the components are as follows:

#### 3.1.1 Backbone network

Backbone network mainly consists of Focus and CSP. Focus module is mainly used to perform slicing operation on the input image to assure adequate feature extraction. In the latest version of YOLOv5s, Focus module has been replaced by a 6×6 convolution operation (C1 module in the **Figure 4**). CSP draws on the structure of Cross Stage Partial Network (CSPNet) (Wang C.-Y. et al., 2020), which has the benefits of improving the learning capability of the network, reducing the model size, breaking the computational bottleneck and solving the problem of gradient information repetition. YOLOv5s has designed two CSP modules, CSP1_X and CSP2_X for the backbone network and the neck, respectively. CSP1 _X consists of Convs (convolution operation + Batch Normalization + SiLu activation function), X residual units (BottleNeck1) and a connection function. We modify the structure of CSP1_X and the proposed attention module CLA is combined with it to form the CLA-CSP1_X module.

#### 3.1.2 SPPF

SPPF consists of multiple Max Pooling layers with kernel size of 5×5 and Convs which enhances feature extraction efficiency by fusing multiple receptive fields of deep feature map. In the AgriPest-YOLO network, this module has been replaced by the proposed GSPPF for further improving the representation of multi-scale features of pests.

#### 3.1.3 Neck

This part mainly consists of Feature Pyramid Network (FPN) and Path Aggregation Network (PAN). PAN adds a new bottom-up pathway to the feature pyramid network to improve the utilization of feature information.

#### 3.1.4 Head

Same as YOLOv4, YOLOv5s has inherited the head structure of YOLOv3. In the training phase, the GIoU loss function (Rezatofighi et al., 2019) as regression loss function of YOLOv5s is used to solve the gradient disappearance problem caused by the non-intersection of the prediction bounding box and the target ground truth. In the testing phase, the traditional NMS method is used to filter the redundant predicted bounding boxes. However, there are a lot of pest overlaps in the dataset, so Soft-NMS has been introduced to replace NMS, reduce the pest miss detection due to overlap.

### 3.2 Coordinate and local attention mechanism

**Figure 5:**
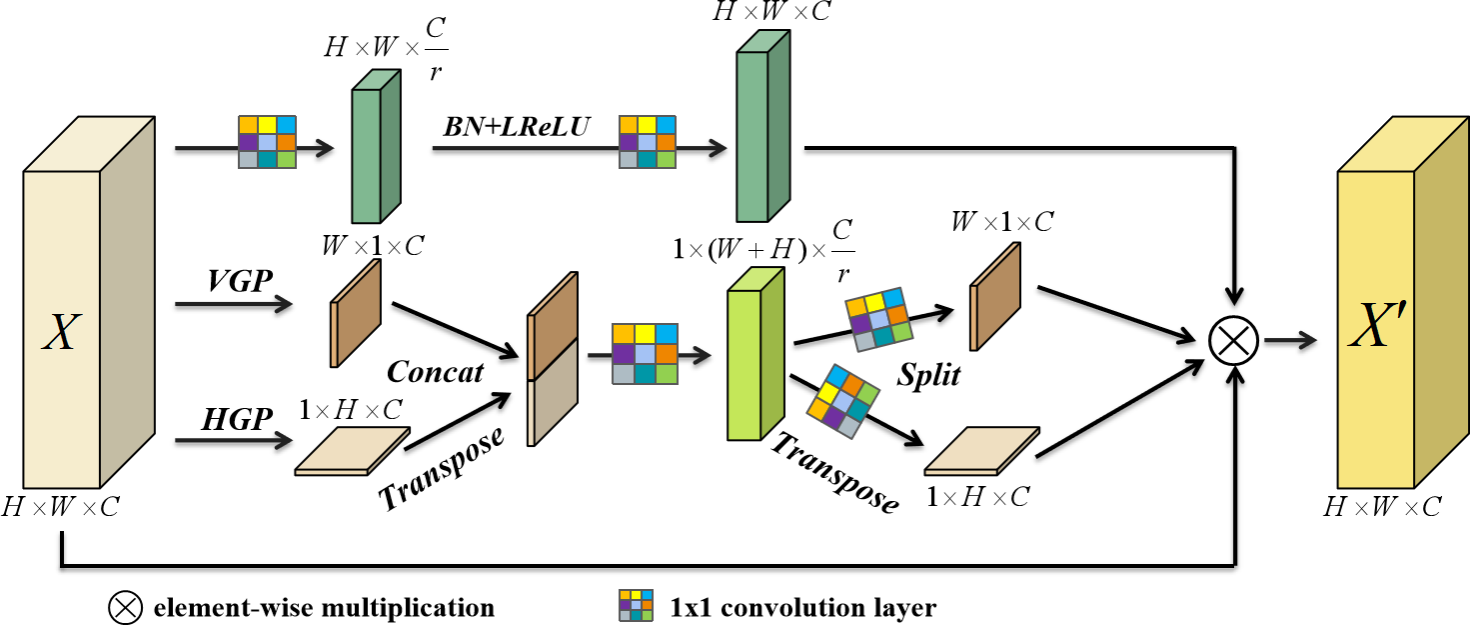
Architecture of CLA module; VGP and HGP are vertical and horizontal pooling operations respectively; BN is Batch Normalization; LReLU is the Leaky ReLu activation function.

Attention mechanism can guide the model to pay attention to the region of interest instead of the whole image. The pest datasets generally have problems such as small size and complex background. Based on the idea of coordinate attention (Hou et al., 2021) and ParseNet (Liu et al., 2015), this paper proposes a novel soft attention mechanism, coordinate and local attention mechanism (CLA), to improve the recognition of these pests by the network. Firstly, the long-term dependencies having location information which are hard to be captured by convolutional operations are modeled by encoding horizontally and vertically, follow by the location information are embedded into the channel attention. And then the local channel attention information is captured. Based on these learnable attention information, the model selectively highlights the valid pest features, filters some noise, contributes to the recalibration of the features, and improves the feature representation of the network. Moreover, the module has a simple structure and generates little computational overhead. The overall architecture of this attention module is shown in **Figure 5**.

Given an intermediate feature map *X*∈*R*^*C*×*H*×*W*^ as input, where the feature map height and width are H,W, respectively, and the channel dimension is C. Firstly, one-dimensional pooling operations with kernel sizes (H,1) and (1, W) are used to encode along the horizontal and vertical directions of the input feature map, respectively, then the output of the c-th channel in height h and width w respectively can be denoted as follows:


(2) $$z_{c}^{h}\left(h\right)=\frac{1}{W}\sum_{0\leq i\leq W}x_{c}\left(h,i\right)$$

(3) $$z_{c}^{w}\left(w\right)=\frac{1}{H}\sum_{0\leq j\leq H}x_{c}\left(j,w\right)$$

where *x*_*c*_ denotes the c-th channel feature map of the intermediate feature map *X* . $z_{c}^{h}\left(h\right)\;$ and $z_{c}^{w}\left(w\right)\;$ denote the different encoding results of *x*_*c*_ , and contain the awareness information of *x*_*c*_ in horizontal and vertical directions, respectively. Then both of the above generated feature maps are aggregated by transpose and concatenate operations, and a feature map $F\in R^{\frac{C}{r}\times \left(H+W\right)}$ , which contains both horizontal and vertical directional awareness information, is generated by convolution operation and nonlinear activation, specifically can be formulated as:


(4) $$F=\delta \left(Conv\left(\left[Z^{h},Z^{w}\right]\right)\right)$$

where *δ* is the nonlinear activation function Leaky ReLu, *Conv* is the convolution operation with convolution kernel parameter of $\frac{C}{r}\times C\times 1\times 1\;$ operation, and […,…] represents the concatenate operation. Then the feature map *F* is split into two different feature tensors, $F^{h}\in R^{\frac{C}{r}\times H}$ and $F^{w}\in R^{\frac{C}{r}\times W}\;$ along the spatial dimension, and the two feature tensor are operated by different kernel convolution operations to obtain attention weights in different directions, *g*^*h*^∈*R*^*C*×*H*×*W*^ and *g*^*w*^∈*R*^*C*×*H*×*W*^ , which are formulated as follows:


(5) $$g^{h}=\sigma \left(Conv\_h\left(F^{h}\right)\right)$$

(6) $$g^{w}=\sigma \left(Conv\_w\left(F^{w}\right)\right)$$

Where *σ* is the sigmoid activation function, *Conv*_*h* and *Conv*_*w* are the convolution kernel parameters as $C\times \frac{C}{r}\times 1\times 1,\;C\times \frac{C}{r}\times 1\times 1$ for the convolution operation.

Next, local attention information is extracted along the channel dimension by local attention consisting of 1×1 convolutional blocks in the CLA module, which helps the network to detect locally distributed tiny pest targets. The local context $x_{c}^{l}\left(i,j\right)$ of the c-th channel feature map can be denoted as:


(7) $$x_{c}^{l}\left(i,j\right)=\delta \left(C2\left(\delta \left(C1\left(x_{c}\left(i,j\right)\right)\right)\right)\right)$$

where C1, C2 are the convolution operations with convolution kernel parameters $\frac{C}{r}\times C\times 1\times 1,\;C\times \frac{C}{r}\times 1\times 1\;$ respectively. Finally, the output feature map *X*^′^ of CLA can be represented as:


(8) $$x_{c}^{'}\left(i,j\right)=x_{c}\left(i,j\right)\times g_{c}^{h}\left(i\right)\times g_{c}^{w}\left(j\right)\times x_{c}^{l}\left(i,j\right)$$

CLA attention module captures long-term dependencies with precise location information and different scale channel attention through the aforementioned behaviors, gains weight coefficients of effective pest features and filters background noise, which can effectively improve the recognition accuracy of pests in complex backgrounds.

### 3.3 Grouping spatial pyramid pooling fast

**Figure 6:**
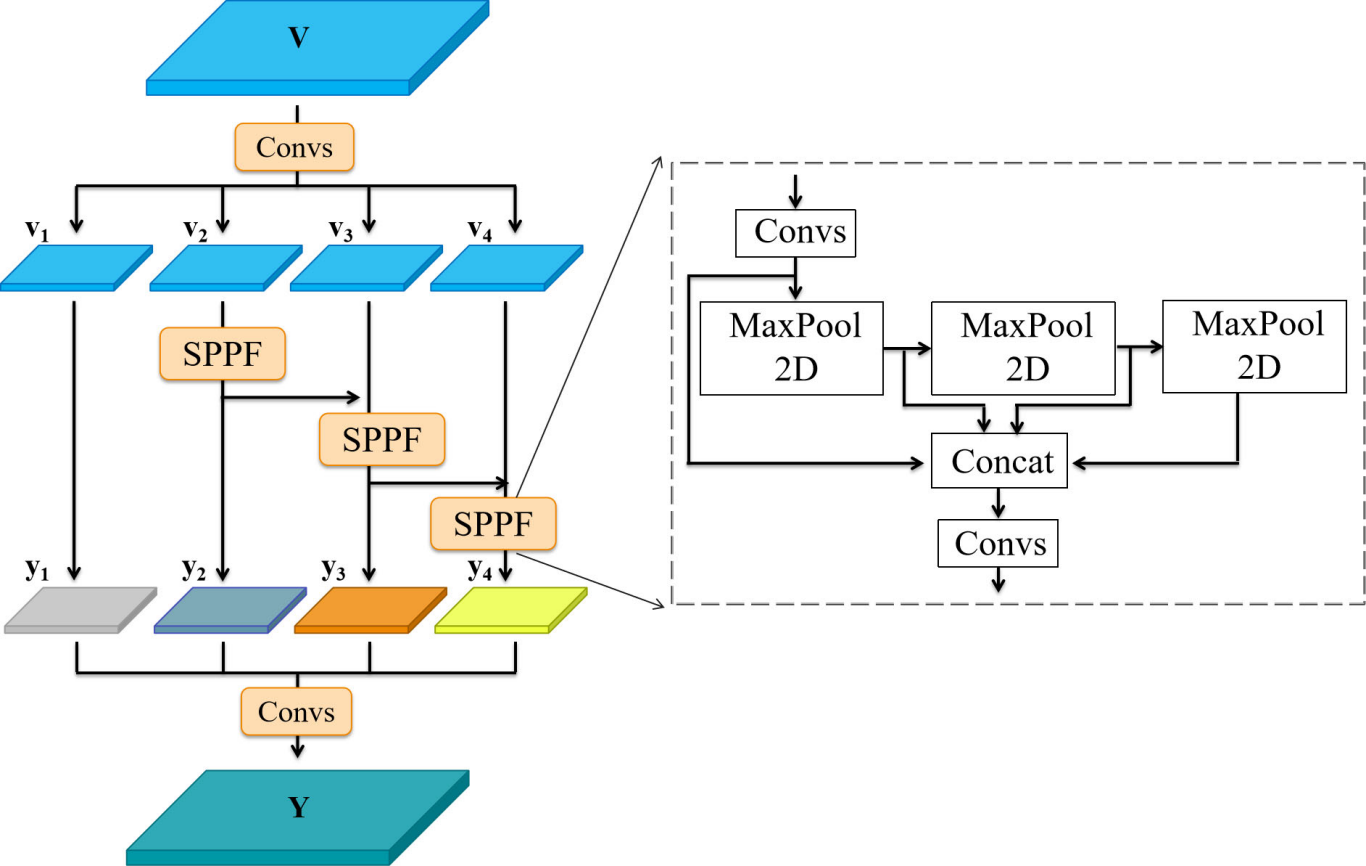
Architecture of GSPPF module, MaxPool2D represents the max pooling operation with the kernel size of 5.


Spatial Pyramid Pooling (SPP) is a common module for YOLOv3,YOLOv4, constructed by four pooling operations with different kernel sizes for extracting salient feature information and improving the classification ability of the model. The spatial pyramid pooling fast (SPPF) in YOLOv5s is an improved version of SPP, which ensures feature information extraction as well as significantly improves computational efficiency, with 277.8% growth compared to SPP. To further intensify the network for multi-scale pest feature extraction, based on the concept of scale dimensionality (Gao et al., 2021), we designed the grouping spatial pyramid pooling fast (GSPPF) instead of SPPF, as shown in **Figure 6**. The details of GSPPF are as follows. For the input deep feature map $v_{i}\in R^{\frac{C}{4}\times H\times W}$ , after passing through the *Convs* block, the input feature map is uniformly divided into four groups, each of which is a subset of the deep feature map $v_{i}\in R^{\frac{C}{4}\times H\times W}$ , where *i*∈{1,2,3,4} . SPPF works on the latter three feature subsets with the purpose of fusing multiple receptive fields, improving the representational power of the feature subsets. The output *y*_*i*−1_ of the previous *SPPF*_*i*−1_ is added to the current subset *v*_*i*_ and then fed into the *SPPF*_*i* _ to get the output *y*_*i*_ of the feature subset. Hence, *y*_*i*_ and the output *Y* of GSPPF can be written as:

(9) $$y_{i}=\{\begin{array}{c} x_{i},\;\;\;\;\;\;\;\;\;i=1\;\\ SPPF_{i}\left(x_{i}\right),\;i=2\\ SPPF_{i}\left(x_{i}+y_{i-1}\right),\;2<i\leq 4 \end{array}$$

(10) $$Y=Convs\left(\left[y_{1},y_{2},y_{3},y_{4}\right]\right)$$

GSPPF has the same position as SPPF in the network, and functions on the deep feature map that contains more semantic information but has lost part of the fine-grained information. The module intensifies the multi-scale representation at a finer-grained level. Through grouping the deep feature maps, each feature subset is treated as an independent feature map with SPPF to extract feature information and expand the receptive fields to achieve feature information reuse and multiple receptive field fusion of features of different scales, which enriches the feature map representation and facilitates the detection of complex multi-target and scale-variant scenarios in the pest dataset.

### 3.4 Final prediction bounding box optimization

In the testing phase, the detector often generates multiple prediction bounding boxes for the same pest object, and the redundant prediction bounding boxes are filtered out in the post-processing phase of YOLOv5s with the NMS approach (Neubeck and Van Gool, 2006): selecting the highest scoring prediction bounding box, and then searching for boxes in the neighborhood that belong to the same category as the highest scoring box but have an overlap greater than a predefined overlap threshold for suppression. However, there is overlap and mutual occlusion between pest targets, which manifests during the prediction stage as the potential presence of multiple pest objects in adjacent prediction bounding boxes. The traditional NMS forces their scores to be zeroed and filtered out, resulting in a large number of misses of pests. To refine the prediction mechanism, we introduced Soft-NMS (Bodla et al., 2017) into the model to improve the detection of overlapping pests. When multiple predicted bounding boxes appear around a pest, their scores are multiplied by a Gaussian function as penalty weights instead of being zeroed directly. The specific calculation is as follows:


(11) $$S_{i}=S_{i}e^{-{\frac{IoU\left(Bbox_{max\;}\;,\;\;bbox_{i}\right)}{\alpha }}^{2}}$$

where *S*_*i*_ is the score of the i-th prediction bounding box, *Bbox*_*max*_ is the prediction bounding box with the highest score, *bbox*_*i*_ is the adjacent bounding box, and the hyperparameter *α* is set to 0.5.

### 3.5 Model evaluation metrics

In the object detection task, the metrics commonly employed to evaluate the accuracy of the model are Precision, Recall, Average Precision (AP) and mean Average Precision (mAP). The specific calculation formula is as follows:


(12) $$Precision=\frac{\#TP}{\#TP+\#FP}$$

(13) $$Recall=\frac{\#TP}{\#TP+\#FN}$$

(14) $$AP=\int_{0}^{1}Precision\;d\;Recall$$

(15) $$AP_{50:95}=\frac{1}{10}\left(AP_{50}+AP_{55}+\ldots +AP_{90}+AP_{95}\right)$$

(16) $$mAP_{@0.5}=\frac{1}{C}\sum_{i=1}^{C}AP^{i}$$

(17) $$mAP_{@\left[0.5:0.95\right]}=\frac{1}{C}\sum_{i=1}^{C}AP_{50:95}^{i}$$

Where TP (true positive) represents the number of correctly detected pest targets, FP (false positive) represents the number of incorrectly detected pest targets, and FN (false negative) represents the number of missed pest targets. *C* is the number of pest categories, which in this paper is 24. For each pest category in the detection, AP is the area under the Precision-Recall curve, *AP*^*i*^ is the AP of the i-th category, mAP0.5 is the average of the AP of all pest categories. *AP*_50_, *AP*_55_,…,*AP*_90_,*AP*_95_ are the mean of Precision under different Recall when taking different IoU thresholds (thresholds from 0.5 to 0.95 with a step size of 0.05), respectively. AP_50:95_ is the average of the ten values of *AP*_50_, *AP*_55_,…,*AP*_90_,*AP*_95_ . mAP_@[0.5:0.95]_ is the average mAP under different IoU thresholds. In agricultural pest detection tasks, for more comprehensive and fairer measurement of pest detection model performance, AP and mAP are usually adopted as the main evaluation metrics. Thus, in this paper, we mainly discuss AP, AP_50:95_, mAP0.5and mAP_@[0.5:0.95]_ as reference metrics.

## 4 Results and discussion

### 4.1 Implementation details

#### 4.1.1 Experiment platform

All experiments in this section were performed on one NVIDIA Tesla V100 GPU with 32G of memory. The software environment is Ubuntu 18.04, Python 3.8, and Pytorch. To accelerate the training process, NVIDIA CUDA10.2 and CUDNN7.6.5 neural network packages were used.

#### 4.1.2 Training settings

The default hyperparameters were set as follows: the initial learning rate was 0.01, the weight decay was 0.0005, and the momentum was set to 0.937. The remaining parameters were adjusted as follows: the batch-size was set to 16, the iteration period (epoch) was set to 300, and the input image size was set to 640×640 . The online data augmentation technique mentioned in Section 2 was used to preprocess the input images during the network training. Furthermore, this paper used transfer learning, based on the pre-training weights obtained from training YOLOv5s on the COCO dataset were used to initialize the model parameters with the aim of equipping the network with fast learning capabilities and generalization (Li H. et al., 2021). Finally, the built-in anchor adjustment function of YOLOv5 was employed to optimize the preset anchor to match pest instances

### 4.2 Comparison with other advanced detectors

To assess the overall performance of the proposed pest detection method, we compared it with several advanced detectors, including classical object detection models: Faster RCNN, Cascade RCNN (Cai and Vasconcelos, 2021), Dynamic RCNN (Zhang et al., 2020), YOLOv4, YOLOX (Ge et al., 2021) and lightweight models: YOLOv4-tiny, Mobilenetv3-YOLOv4, YOLOv5s. Among them, Mobilenetv3-YOLOv4 is a lightweight version of YOLOv4 with the backbone replaced by Mobilenetv3 (Howard et al., 2019), and YOLOv4-tiny is a simplified version of YOLOv4. All comparison models have the same experimental environment and the parameters are consistent with the original settings in order to ensure the credibility of the results. It is worth noting that when selecting the backbone network for the two-stage comparison model, we considered deeper backbone networks, such as ResNet101 (He et al., 2016). However, the small size of pests in the dataset contains less feature information, and deepening the network layers has no benefits in recognizing tiny pests, but increases the computational burden, so ResNet50 was selected as the backbone of the two-stage comparison model. The results of the quantitative comparison are reported in **Table 2**, it can be seen that the proposed method outperforms the other advanced detectors. From the concrete evaluation metrics, AgriPest-YOLO achieves 71.3% mAP_@0.5_, 3.5% improvement compared to YOLOv5s, 10.2% improvement compared to Cascade RCNN, the best performing model in the two-stage models. Moreover, to further investigate the localization performance of AgriPest-YOLO in pest detection, mAP_@[0.5:0.95]_ of each model calculated at different thresholds are given in **Table 2**. The results in the table show that AgriPest-YOLO still outperforms the detection results of other models at more stringent IoU, such as compared with YOLOv5s, YOLOv4 and Cascade RCNN, respectively, improved 5%, 4.1%, and 8.5%, which indicates that AgriPest-YOLO has less localization error for pest targets.

**Table 2:** Comparison of pest detection results between different models.

Models	Faster RCNN	Cascade RCNN	Dynamic RCNN	YOLOX	YOLOv4	YOLOv4-tiny	YOLOv4	YOLOv5s	AgriPest-YOLO
Backbone	ResNet50-FPN	ResNet50-FPN	ResNet50-FPN	CSPDarknet53	CSPDarknet53	CSPDarknet53-tiny	Mobilenetv3	\	\
mAP_@0.5_(%)	59.4	61.1	59.1	51.4	68.2	53.34	55.49	67.8	71.3
mAP_@[0.5:0.95]_(%)	36.47	38.4	36.84	31.88	42.8	28.5	30.6	41.9	46.9

The goal of this paper is proposed a real-time lightweight detection model for achieving a sound balance between efficiency, accuracy and model size for agricultural pest detection. Hence, not only the accuracy of detection but also its size and detection speed should be discussed when evaluating the pest detection model. We compare GFLOPs, parameters, model size, and inference time of AgriPest-YOLO with other detectors (the lightweight model and the better performing classical object detection model in **Table 2**). For the fairness of the comparison experiments, all detectors are run on the same NVIDIA TeslaV100 GPU, and the input image size is set to 640 × 640 . The experimental results are shown in **Table 3**, from which it is observed that our approach achieves 8.8ms inference time, 16.2 GFLOPs, 7.35 MB number of parameters and 15.1 MB model size, with only a minor increase in computational overhead. Compared with the YOLOv4 model in **Table 2**, which has sub-optimal detection results, the inference time is reduced by half, and GFLOPs, number of parameters and model size are reduced to 11.42%, 11.47 and 5.86%, respectively, the proposed method has a significant superiority in terms of detection efficiency. Compared to the other five comparison models, only inferior to the lightweight models YOLOv4-tiny and YOLOv5s. However, the detection accuracy achieved with our method obviously exceeds them. In summary, AgriPest-YOLO has shown good results in several aspects in pest detection tasks, while considering the lightweight to ensure the detection accuracy and speed, the overall performance is more superior and suitable for real-time pest detection tasks in practical conditions.

**Table 3:** Comparison of pest detection efficiency between different models.

Models	GFLOPs	Parameters (MB)	Model size (MB)	Inference time (ms)
Faster RCNN	206.8	41.24	331.2	40
Cascade RCNN	234.6	69.0	553.3	49.5
YOLOv4	141.8	64.06	257.7	17.7
YOLOv4-tiny	16.2	5.93	22.6	4.44
Mobilenetv3-YOLOv4	17.0	11.43	56.9	11.2
YOLOv5s	16.0	7.08	14.5	6.1
AgriPest-YOLO	16.2	7.35	15.1	8.8

GFLOPS indicates Giga Floating-point Operations Per Second; Parameter indicates the total number of parameters of the model; Inference time indicates the inference time on one single image.

### 4.3 Comparison of detection performance on hard-to-detect pests

**Figure 7:**
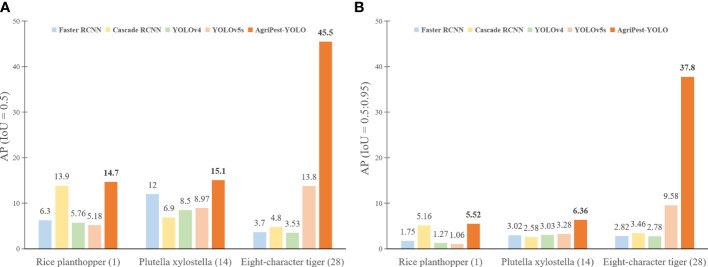
Detection results of different models for pests with few samples and extremely small pests. **(A, B)** illustrate the detection results under different IoU thresholds respectively.

In the field of multi-class agricultural pest detection, the pest size and the number of instances present in the dataset have a great impact on the model performance. Extreme small pests and unbalanced distribution of pest categories are the main attributes of Pest24, presenting new challenges in deep learning-based object detection (Wang Q.-J. et al., 2020). These hard-to-detect pests are mainly separated into three categories, Rice planthopper (index 1), Plutella xylostella (index 14) and Eightcharacter tiger (index 28), where Rice planthopper and Plutella xylostella have very small sizes, with relative sizes of only 0.034 and 0.043, and the number of instances of Eightcharacter tiger is very few with only 154, much lower than the average number of category instances. To analyze the detection performance of AgriPest-YOLO on pests falling into few samples and tiny pests, these three categories of hard-to-detect pests were selected for validation, and the results are shown in **Figure 7**. This figure shows that for the extreme small pests, Rice planthopper and Plutella xylostella, there is still poor detection accuracy for them, especially for the one-stage models YOLOv4, YOLOv5s, which have the advantage in detection speed but the recognition of pests is less than the two-stage RCNN series models. In contrast, AgriPest-YOLO, benefiting from attention mechanism and GSPPF module, has improved the ability to represent more small pest features, and the performance on extreme small pests is also better than other advanced models, especially compared with the same one-stage YOLO series models. For the few samples pest Eightcharacter tige, AgriPest-YOLO performs well, achieving 45.5% AP, which exceeds by far the other comparison models. This demonstrates that our method can extract much more effective pest features in the limited number of samples, resulting in better fitting of the network during training. However, **Figure 7B** shows that higher required IoU thresholds result in significantly reduced detection performance on these hard-to-detect pests, especially for extreme small pests. This indicates the lack of ability of models to produce precisely predicted bounding boxes for extreme small pests, and these will be improved in future work.

### 4.4 Comparison of detection performance of pests in densely distributed and complex backgrounds

**Figure 8:**
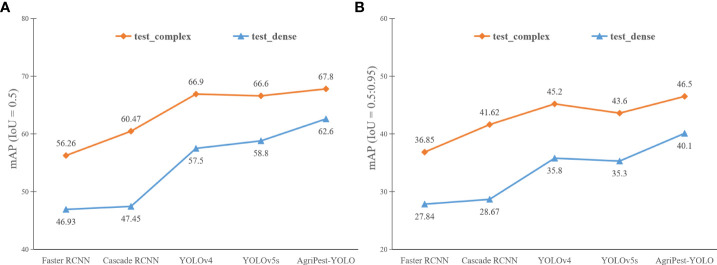
Detection results of different models for pests in densely distributed and complex backgrounds. Test_complex is the test set for complex background pests and test_dense is the test set for densely distributed pests. **(A, B)** illustrate the detection results under different IoU thresholds respectively.

**Figure 9:**
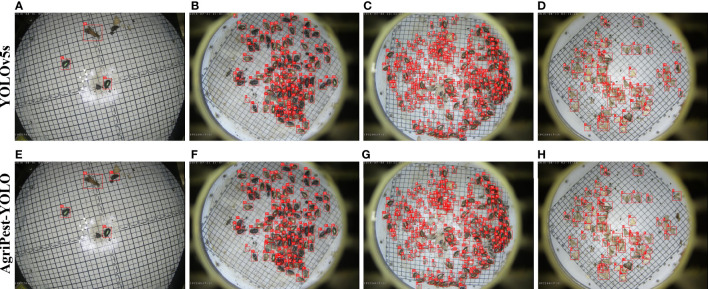
Detection results of YOLOv5s and the proposed AgriPest-YOLO. **(A, D, E, H)** belongs to the test_complex test set, **(B, C, F, G)** belongs to the test_dense test set.

In the Pest24 dataset, the dense distribution of pests and the multiple complex backgrounds described in Section 2 are critical factors that affect the detection performance. To investigate their impact, we split the test set of the Pest24 dataset into two test subsets, including the manually selected test set, test_dense, consisting of 500 samples with dense distribution of pests and the test set, test_complex, consisting of 450 samples with complex backgrounds. Note that the two factors of dense distribution and complex background may not necessarily exist independently, and may contain both dense distribution and multiple complex backgrounds in a single image. Experimental results are shown in **Figure 8**. It can be seen that the one-stage YOLO series algorithms are more advantageous in complex background or densely distributed pest detection, and AgriPest-YOLO is the best performer among the YOLO series, especially in detecting densely distributed pests, as shown in **Figure 9G**, AgriPest-YOLO can recognize more of the correct target pests. For pest detection in complex backgrounds, it can be seen in **Figure 8B** that our proposed method still performs well under stricter thresholds and can provide more accurate localization information. As shown in **Figures 9A, E**, AgriPest-YOLO hit more pest targets compared to YOLOv5s in the complex background, more importantly predicting finer bounding box coordinates.

### 4.5 Visualization of detection results

In this section, we visualize part of the pest detection results to directly observe the strengths of our proposed pest detection method, as shown in **Figure 9**. **Table 4** presents the number of real pest instances in each detection result image, the corresponding detection results for the detected, undetected and misdetected pests of different models. Following the results in the figure and table, it can be found *via* quantitative and qualitative analysis that the improved model performs well in detecting pests with sparse or dense distribution compared to the original YOLOv5s. When noises (non-target pests with similar appearance) were present in the images, as shown in **Figures 9D, H**, AgriPest-YOLO presented better robustness as the attention module emphasized the effective pest features and filtered out other interferences. When images with dense distribution of small pests are present, as shown in **Figures 9C, G**, AgriPest-YOLO has more pests detected and fewer misdetection, which will be helpful in the future for forecasting infestation trends by counting different classes of pests.

**Table 4:** Detection results between YOLOv5s and AgriPest-YOLO for pests as shown in **Figure 9**.

Figs.No	Total number of pests	YOLOv5s			AgriPest-YOLO		
		Detected	Undetected	Misdetection	Detected	Undetected	Misdetection
**Figures 9A, E**	4	3	1	1	4	4	/
**Figures 9B, F**	95	83	12	20	90	5	13
**Figures 9C, G**	214	177	37	84	186	28	58
**Figures 9D, H**	54	44	10	26	49	5	13

### 4.6 Ablation study

#### 4.6.1 Impact of the proposed module on detection performance

Our proposed pest detector based on YOLOv5s model contributes four elements, including the attention and GSPPF modules developed, the introduction of Soft-NMS as a post-processing method, and online data augmentation methods to enrich the samples. To further investigate the impact of each element on pest detection, the results of the ablation study are given in **Table 5**. First, the data augmentation method improved pest detection result dramatically because online data augmentation enriched the diversity of training data, which facilitated small target pest detection. Then, the introduced Soft-NMS working on the prediction layer reduced the missed detection from overlapping pests and improved the mAP0.5by 0.9%. Finally, both the proposed CLA and GSPP presented the positive impact in the pest detection results, improving the performance to 71.3%. Besides, as seen from the mAP_@[0.5:0.95]_ computed at higher thresholds, the proposed methods contribute to the generation of high-quality bounding boxes.

**Table 5:** The impact of each major element of the proposed pest detection model on detection results.

YOLOv5s	Data augmentation	Soft-NMS	GSPPF	CLA	mAP_@0.5_(%)	mAP_@[0.5:0.95]_(%)
√					52.7	30.3
√	√				67.8	41.9
√	√	√			68.7	43.8
√	√	√	√		70.6	45.6
√	√	√	√	√	71.3	46.9

The check mark indicates that the method in the same column has been selected.

#### 4.6.2 Impact of different activation functions in the attention module

Considering the special properties of pest detection tasks such as small targets, dense distribution, and complex backgrounds, it is necessary for the attention module to select the suitable activation functions for optimal detection performance. We selected several state-of-the-art activation functions including ReLu6, Mish, SiLu, and Leaky ReLu, and conducted multiple control experiments to investigate their impact on the pest detection task. Experimental results are reported in **Table 6**. It can be found that Leaky ReLu activation function has the best performance because it effectively addresses the gradient disappearance problem in the dense object detection task and maximizes the weight retention, to achieve the model performance improvement. Moreover, the table gives a comparison of the results of two attention mechanisms, coordinate attention (CA) and CLA, on the pest detection task, where our proposed CLA is more applicable in the pest detection task due to the complement of local attention information when the parameters are the same.

**Table 6:** The impact of different activation functions in attention mechanism on model performance.

Attention mechanism	CA	CLA	CLA	CLA	CLA
Activation function	ReLu6	ReLu6	Mish	SiLu	Leaky ReLu
mAP_@0.5_(%)	68.1	68.7	68.3	68.9	69.1
mAP_@[0.5:0.95]_(%)	42.0	42.5	42.3	42.3	42.8

#### 4.6.3 Impact of different data augmentation methods on detection performance

To enrich the diversity in the training samples in the Pest24 dataset and to improve the robustness of the detector, five online data augmentation methods were adopted, including HSV, Filp, Translate, Scale, and Mosaic. We performed multiple comparison experiments using the control variables method, adding one data augmentation method to the training model at a time, to validate the impact of different augmentation methods for the multi-category pest detection task. The results are shown in **Table 7**, where all data augmentation methods demonstrate the positive impact on improving the pest detector performance.

**Table 7:** The impact of different data augmentation methods on pest detection results.

HSV	Flip	Translate	Scale	Mosaic	mAP@0.5(%)	mAP@[0.5:0.95](%)
					52.7	30.3
√					53.2	30.1
√	√				59.3	34.7
√	√	√			63.1	37.8
√	√	√	√		66.7	41.3
√	√	√	√	√	67.8	41.9

The check mark indicates that the method in the same column has been selected.

### 4.7 Comparison of the robustness of detection with different noise

**Figure 10:**
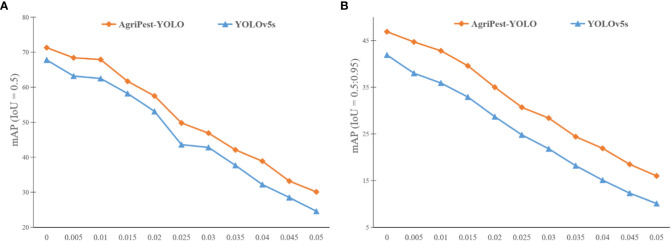
Detection results of YOLOv5s and the proposed AgriPest-YOLO at different noise levels. The horizontal axis indicates the noise intensity added to the test set images and the vertical axis indicates the detection accuracy of the models. **(A, B)** illustrate the detection results under different IoU thresholds respectively.

To further investigate the quality of AgriPest-YOLO, we have added different levels of Salt & Pepper noise and Gaussian noise in all images of the test set and analyzed the detection results to evaluate the robustness of AgriPest-YOLO. The Salt & Pepper noise will generate random white or black dots on the images. The noise level ranges from 0.005 to 0.05 in steps of 0.005. In addition, the mean value of Gaussian noise is 0.1 with standard deviation of 0.05. Experimental results are shown in **Figure 10**. Obviously, as the noise intensity rises, the detection accuracy of the models decreases, because the noise has strong effects on small pest detection and it is hard for the model to extract enough pest features in strong noise. In addition, the detection accuracy of AgriPest-YOLO is always higher than that of YOLOv5s for different levels of noise interference, and the accuracy gap in the presence of noise interference is higher than that in the absence of noise interference. Therefore, we can conclude that the improved model, AgriPest-YOLO, has better noise immunity and robustness.

## 5 Conclusion

To address the limitations of multi-class agricultural pest detection from light-trap images, this work proposes a lightweight pest detection method called Agripest-YOLO. As part of our proposal, a new attention mechanism was designed that can improve pest feature extraction, filter out worthless features, which can augment the detection performance of pests in complex backgrounds. Then, GSPPF was developed to represent multi-scale features of pests at a finer granularity level, achieve feature reuse and multiple fusion of pest features of different scales, enrich multi-scale representation of pest features, and thereby obtain better multiple scales pest detection performance. Experimental results show that AgriPest-YOLO outperforms other advanced methods in several aspects, improving the detection accuracy while considering lightweight and detection speed. Furthermore, AgriPest-YOLO has the advantage of recognizing densely distributed and complex background pests. And it has broad application prospects due to its lightweight design. However, AgriPest-YOLO still has limitations, such as for the extreme small pest, Rice planthopper (index 1) and Plutella xylostella (index 14), although it has been optimized, the detection accuracy remains poor. In the future work, we will try to further address the problem of extreme small pest identification and localization.

## Data availability statement

The original contributions presented in the study are included in the article/Supplementary Material. Further inquiries can be directed to the corresponding authors.

## Author contributions

WZ: methodology, conceptualization, software, investigation, and writing draft. HH: supervision, writing and revising. YS: supervision, writing and revising. XW: validation. All authors contributed to the article and approved the submitted version.
